# HER-2 ultra-low breast cancer: exploring the clinicopathological features and prognosis in a retrospective study

**DOI:** 10.3389/fonc.2023.1210314

**Published:** 2023-08-17

**Authors:** Jiajie Shi, Liqiu Zhang, Cuizhi Geng

**Affiliations:** Department of Breast Oncology, Fourth Hospital of Hebei Medical University, Shijiazhuang, China

**Keywords:** HER-2 ultra-low expression, breast cancer, disease-free survival, overall survival, clinicopathological features

## Abstract

**Objective:**

To explore the clinicopathological features of patients with ultra-low expression of human epidermal growth factor 2 (HER-2) in breast cancer and its impact on prognosis

**Methods:**

Data from 1024 patients with primary breast cancer having HER-2 ultra-low expression from January 01, 2018, to December 31, 2018, were collected and analyzed retrospectively. The clinicopathological features and prognosis were compared using a chi-squared test or Fisher exact probability method. COX regression analysis and log-rank test were used to explore the factors related to the postoperative 5-year survival rate. All analytical data were defined as statistically significant (*P* < 0.05).

**Results:**

Overall survival (OS) was higher in the HER-2 ultra-low group compared to the low expression group (*P* = 0.022). The tumor diameter, lymph node metastasis (LNM), and Ki67 expression were factors affecting DFS in the HER-2 ultra-low expression group (*P* < 0.05). The tumor diameter and LNM were risk factors affecting the OS (*P* < 0.05) in the HER-2 ultra-low expression group. LNM and Ki67 expression were risk factors affecting DFS (*P* < 0.05) in the HER-2 low expression group. LNM was considered an independent risk factor affecting OS (*P* < 0.05).

**Conclusion:**

Breast cancer with HER-2 ultra-low expression has differences in the clinicopathological features. Breast cancer with HER-2 low expression is more aggressive and has a worse prognosis. This study provides a reference to consider in the treatment of HER-2-low and -ultra-low expression breast cancer.

## Introduction

Breast cancer is currently the leading malignant cancer diagnosed among women, with incidences accounting for about 11.7% of the total number of new cases of cancer, as per the updated Global Cancer statistics of the International Agency for Research on Cancer (IARC) for 2020. Although the diagnosis and treatment strategies of breast cancer are constantly evolving with advances cited in The Times, the mortality rate remains high at 6.9% among all cancer deaths (1). The prognosis among breast cancer patients varies based on the molecular subtypes; therefore, precision medicine has currently gained increased attention.

Based on the pathology, experts at the 2013 St. Gallen International Breast Cancer Conference have categorized breast cancer into four molecular subtypes, viz. Luminal A, Luminal B, triple-negative (TNBC), and human epidermal growth factor receptor 2 positive (HER-2+) (2). HER-2 is an important driver gene for breast cancer and shows tyrosine kinase activity. It is also an important predictor of prognosis. Its expression levels are indicative of the efficacy of anti-HER-2 therapeutic drugs in breast cancer. Overexpression of HER-2 is associated with highly aggressive tumors and poor prognosis (3). With the availability of broad range anti-HER-2 drugs, such as trastuzumab, lapatinib, pertuzumab, pyrotinib, and trastuzumab emtansine (T-DM1), the prognosis among patients with HER-2+ early and advanced breast cancer has significantly improved (4).

In recent years, the focus on breast cancer with HER-2-low expression has increased with evolving research on decitabine, a novel antibody-drug conjugate (ADC) drug. According to the testing guidelines of the 2022 American Society of Clinical Oncology/College of American Pathologists (ASCO/CAP), HER-2 low expression is defined as a breast cancer subgroup having immunohistochemistry (IHC) score of 1+ or 2+ and negative for fluorescence *in situ* hybridization (FISH−) test (5). Previously, patients with HER-2 low expression were often classified as having HER-2− breast cancer. Further, patients with HER-2 low expression were treated with anti-HER-2 drugs, such as trastuzumab and T-DM1. In the DESTINY-Breast04 study involving patients with advanced breast cancer and HER-2 low expression who had previously received first- or second-line hormone receptor-positive (HR+) endocrine therapy, trastuzumab deruxtecan (T-DXd) significantly improved the progression-free survival (PFS) and overall survival (OS) compared with physician-selected chemotherapy (median PFS [mPFS], 10.1 months vs 5.4 months, P < 0.05). The median OS was 23.9 months versus 17.5 months. T-DXd showed a favorable therapeutic effect and survival benefit in HER-2-low expression cases (6). However, in HER-2− breast cancer, some breast cancer cells expressed different levels of HER-2 on the surface. The DAISY study (NCT04132960) enrolled patients with HER-2 non-expression, including those with HER-2 ultra-low expression. Ultra-low expression is defined as a subgroup having an IHC score close to 1+, but not completely HER-2− (faint staining in ≤10% cells). An objective response rate (ORR) seen with T-DXd drugs warrants the re-review and reclassification of the concept of HER-2 low breast cancer (7). The recent DESTINY-Breast06 study further explores the efficacy and safety of T-DXd in patients with HER-2 low and ultra-low expression and HR+ advanced breast cancer refractory to endocrine therapy and without chemotherapy. The data of this study are expected to increase the understanding of the diagnosis and treatment landscape in HER-2 ultra-low expression (8). Our retrospective analysis aimed to understand whether the clinicopathological features and prognosis of patients with HER-2 low and ultra-low subtypes are similar.

In this regard, we retrospectively analyzed and compared the clinicopathological characteristics along with prognosis among patients with HER-2 low and ultra-low expression breast cancer. With this study, we hope to provide a basis for a better clinical treatment landscape of breast cancer patients with HER-2 ultra-low expression.

## Materials and methods

### Patient data

Data of patients with HER-2 ultra-low or low expression breast cancer who underwent surgery in the Breast Center of the Fourth Hospital of Hebei Medical University in China between January 1, 2018, and December 31, 2018, were collected for follow-up and analysis.

The key inclusion criteria for selecting the cases were (1) confirmed pathological diagnosis of invasive breast cancer; (2) measurable tumor diameter using ultrasound or magnetic resonance imaging; (3) HER-2 low or ultra-low expression confirmed by IHC; and (4) availability of complete follow-up and clinicopathological data. The key criteria for excluding cases were (1) males with breast cancer; (2) receiving neoadjuvant chemotherapy or endocrine therapy prior to surgery; (3) distant metastasis or other malignant tumors found during general examination prior to surgery; (4) synchronous or metachronous bilateral breast cancer.

All patients had undergone breast ultrasound, mammography, and bilateral breast-enhanced magnetic resonance imaging prior to surgery to evaluate the primary tumor and lymph node status. Computed tomography and whole-body bone radionuclide computed tomography was done to exclude distant metastasis or other malignant tumors.

### Tumor characteristics

Data on disease pathology for all patients were obtained from the Department of Pathology of our hospital. The estrogen receptor-positive (ER+) status of tumors was defined as positive IHC staining of >1% cells. Further, the ER positivity between 1% and 9% was defined as ER-weakly positive (ER-low). The progesterone receptor positive (PR+) status was defined as positive IHC staining of >20% of cells. ER- or PR-positive was defined as HR-positive. The HER-2+ status was defined as having an IHC score of 3+ or 2+ and FISH+; HER-2 low expression was defined as having an IHC score of 1+ or 2+ and FISH−; and HER-2− was defined as having an IHC score of 0. HER-2− negative cells showed no HER-2 protein staining, or ≤10% of invasive cells showed incomplete weak cell membrane staining. TNBC was defined as having an IHC score of 0 and FISH−. In this study, HER-2 ultra-low expression was defined as invasive cells showing at least ≤10% membrane protein staining or incomplete weak membrane staining. Ki67 is a nuclear protein associated with cell proliferation. Low Ki67 expression was defined as a score of ≤30% and high Ki67 expression as a score of >30%. The T stage was categorized based on tumor size as T1 (<5 cm), T2 (> 2 ≤5 cm), T3 (> 5 cm), and T4 (any size with direct extension to the chest wall or skin). The axillary lymph node stage was categorized as N0 (0+ axillary lymph nodes), N1 (metastasis to 1–3 axillary lymph nodes), N2 (metastasis to 4–9 axillary lymph nodes), and N3 (metastasis to ≥10 axillary lymph nodes). Metastasis was categorized as M0 (no clinical or radiographic evidence of distant metastasis) and M1 (clinical or radiographic evidence of distant metastasis with histologically confirmed metastases of >2 mm). The histological type was determined according to the World Health Organization classification (5).

### Follow-up and outcome variables

Patients were followed up through outpatient review, hospitalization, and telephone. The follow-up began on the first postoperative day and ended on January 31, 2023, or death, whichever came first. Disease-free survival (DFS) was defined as the time from postoperative day 1 to the first detection of recurrence and/or metastasis. OS was defined as the time from postoperative day 1 to death or the end of follow-up, whichever came first.

### Statistical analyses

The follow-up data were grouped to establish a database. The Statistical Package for the Social Sciences (SPSS) software, Version 26.0, was used for statistical analyses. The Chi-square test or Fisher’s exact test was used to compare the clinicopathological characteristics, recurrence, metastasis, and survival between Her-2 low and ultra-low groups. A univariate COX regression analysis was used to analyze the influence of related factors on the 5-year survival rate of patients after surgery. The Kaplan–Meier method was used to generate the survival curve, and a log-rank test was used to compare the prognosis between the two groups. COX regression was used for multivariate analysis. All analyzed data were presented as *P* < 0.05, which was defined as statistically significant.

## Results

### Patient characteristics

A total of 1024 patients who underwent breast cancer surgery in the Breast Center of the Fourth Hospital of Hebei Medical University from January 1, 2018, to December 31, 2018, met the inclusion criteria. Among them, 27 patients were lost to follow-up, with a loss rate of 2.7%. The follow-up duration ranged from 4 months to 61 months, and the median follow-up duration was 53 months. HER-2 ultra-low expression was seen in 249 patients (24.9%) and HER-2 low expression in 748 patients (75.1%). Local recurrence or distant metastasis was reported in 29 cases (11.6%) in the HER-2 ultra-low group and in 60 cases (8.0%) in the HER-2 low expression group. In the HER-2 ultra-low expression group, 17 (6.8%) deaths due to local recurrence or distant metastasis and 2 (0.8%) deaths due to other causes were reported. In the HER-2 low expression group, 26 (3.4%) deaths due to local recurrence or distant metastasis, and 7 (0.9%) due to other causes were reported.

### Analysis of clinicopathological characteristics of HER-2 ultra-low and low expression groups

The age range was 28-79 years (median, 53 years) and 25-86 years (median, 55 years) for the HER-2 ultra-low and low groups, respectively. The age parameters were divided into two groups <55 years and ≥55 years. In the HER-2 ultra-low group, 145 (58.2%) cases were younger than 55 years. In the HER-2 low group, 372 (39.7%) cases were younger than 55 years old. Compared with the HER-2 low group, the age of onset for patients in the HER-2 ultra-low group was lower (P < 0.05). In the HER-2 ultra-low group, 123 (49.4%) cases were categorized as having T1 tumor size, 116 (46.6%) cases as T2, 8 (3.2%) cases as T3; and 2 (0.8%) cases as T4. In the HER-2 low group, 285 (38.1%) cases were categorized as T1 tumor size, 404 (54.1%) cases as T2, 59 (5.7%) cases as T3, and 16 (2.1%) cases as T4. The HER-2 ultra-low group included patients who have a smaller tumor diameter and a high proportion of early postoperative T stage compared to the HER-2 low group (P < 0.05). The HER-2 ultra-low group had 113 (45.4%) cases of pTNM stage I, 113 (45.4%) cases of stage II, and 23 (9.2%) cases of stage III. The HER-2 low group included 265 (35.4%) cases of stage I and 397 (53.1%) cases of stage II. The proportion of early pTNM stage cases was higher in the ultra-low group than that in the low group (P < 0.05). In the HER-2 ultra-low group, 9 (3.6%) cases were histopathological grade I, 112 (45%) cases were grade II, 81 (32.5%) cases were grade III, and 47 (18.9%) cases had other histological grades. In the HER-2 low group, 54 (7.2%) were grade I, 466 (62.3%) cases were grade II, 111 (14.8%) cases were grade III, and 117 (15.6%) cases had other histological grades. The proportion of histological grade III cases in the HER-2 ultra-low group was significantly higher than that in the HER-2 low group (P < 0.001). High p53 expression was seen in 77 (30.9%) cases in the HER-2 ultra-low group and in 117 (15.6%) cases in the HER-2 low group. The expression intensity of p53 in the HER-2 ultra-low group was higher (P < 0.001). Further, 138 (55.4%) cases had high type II topoisomerase (TOPOII) expression in the HER-2 ultra-low group, whereas 320 (42.8%) cases had high TOPOII expression in the HER-2 low group. The expression intensity of TOPOII in the ultra-low group was higher (P < 0.05). High Ki67 expression was seen in 167 (67.1%) cases in the HER-2 ultra-low group and in 407 (54.4%) cases in the HER-2 low group. The expression intensity of Ki67 in the HER-2 ultra-low group was higher (P < 0.001). In the HER-2 ultra-low group, 182 (73.1%) cases were ER+ and 161 (67.4%) cases were PR+. In the HER-2 low group, 672 (89.2%) cases were ER+ and 605 (80.9%) cases were PR+. The positivity rates of ER and PR in the HER-2 ultra-low group were significantly lower than those in the low group (P < 0.001). However, there was no significant difference in lymph node metastasis between the two groups (P = 0.736; **Table 1**). The type of surgery is also related to the HER-2 expression. The number of cases that underwent breast-conserving surgery with axillary lymph node dissection (ALND, 3.2%) and breast-conserving surgery with sentinel lymph node biopsy (SLNB, 31.3%) in the HER-2 ultra-low group was significantly higher compared to the HER-2 low group (breast-conserving surgery+ALND, 1.6%; breast-conserving surgery+SLNB, 17.1%). The number of cases that underwent mastectomy with SLNB (40.1%) and modified radical mastectomy (41.2%) in the HER-2 low group was significantly higher compared to the HER-2 ultra-low group (mastectomy+SLNB, 34.9%; modified radical mastectomy, 30.5%).

**Table 1:** Clinical and pathological features of the HER-2 ultra-low and HER-2 low expression groups.

Characteristics	HER-2 ultra-low, n (%)	HER-2 low, n (%)	*P*
Age (y)			0.02
≤55	145 (58.2%)	372 (49.7%)	
≥55	104 (41.8%)	376 (50.3%)	
T stage			0.007
1	123 (49.4%)	285 (38.1%)	
2	116 (46.6%)	404 (54.1%)	
3	8 (3.2%)	59 (5.7%)	
4	2 (0.8%)	16 (2.1%)	
N stage			0.736
0	167 (67.1%)	482 (64.5%)	
1	63 (25.3%)	200 (26.7%)	
2	14 (5.6%)	42 (5.6%)	
3	5 (2.0%)	24 (3.2%)	
Histological stage			<0.001
I	9 (3.6%)	54 (7.2%)	
II	112 (45.0%)	466 (62.3%)	
III	81 (32.5%)	111 (14.8%)	
Others	47 (18.9%)	117 (15.6%)	
pTNM			
I	113 (45.4%)	265 (35.4%)	0.019
II	113 (45.4%)	397 (53.1%)	
III	23 (9.2%)	86 (11.5%)	
P53			<0.001
≤10%	172 (69.1%)	631 (84.4%)	
≥10%	77 (30.9%)	117 (15.6%)	
TOPOII			0.001
≤20%	111 (44.6%)	428 (57.2%)	
≥20%	138 (55.4%)	320 (42.8%)	
Ki67			<0.001
≤30%	82 (32.9%)	341 (45.6%)	
≥30%	167 (67.1%)	407 (54.4%)	
ER			<0.001
Positive	182 (73.1%)	672 (89.8%)	
Negative	67 (26.9%)	76 (10.2%)	
PR			<0.001
Positive	161 (67.4%)	605 (80.9%)	
Negative	88 (35.3%)	143 (19.1%)	
Surgery type			<0.001
Breast-conserving surgery+ALND	8 (3.2%)	12 (1.6%)	
Breast-conserving surgery+SLNB	78 (31.3%)	128 (17.1%)	
Mastectomy+SLNB	87 (34.9%)	300 (40.1%)	
Modified radical mastectomy	76 (30.5%)	308 (41.2%)	

SLNB, sentinel lymph node biopsy; ALND, axillary lymph node dissection.

### Analysis of prognosis in the HER-2 ultra-low and low expression groups

Local recurrence or distant metastasis was reported in 29 (11.6%) cases in the HER-2 ultra-low group and in 60 (8%) cases in the HER-2 low group. There was no significant difference in the 5-year recurrence or metastasis rate between the HER-2 ultra-low and low groups (11.6% vs 8.0; P = 0.082; **Table 2**).

**Table 2:** Relationship between HER-2 expression intensity and prognosis.

Recurrence/metastasis	HER-2 ultra-low, n (%)	HER-2 low, n (%)	*P*
Total			0.082
Yes	29 (11.6)	60 (8.0)	
No	22 (88.4)	688 (92.0)	
TNBC subgroup			0.195
Yes	11 (16.4)	7 (9.2)	
No	56 (83.6)	69 (90.8)	
HR-positive subgroup			0.385
Yes	18 (9.9)	53 (7.9)	
No	164 (90.1)	619 (92.1)	

For the triple-negative breast cancer (TNBC) subgroup, recurrence and metastasis events were seen in 11 (16.4%) cases in the HER-2 ultra-low group and 7 (9.2%) cases in the HER-2 low group. In the HR+ subgroup, recurrence and metastasis events were seen in 18 (9.9%) cases with HER-2 low expression and in 53 (7.9%) cases with HER-2 ultra-low expression. A subgroup analysis showed that there was no significant difference in recurrence or metastasis between the HER-2 ultra-low and low groups within the TNBC and HR+ subgroups (P < 0.05; **Table 2**).

**Figure 1:**
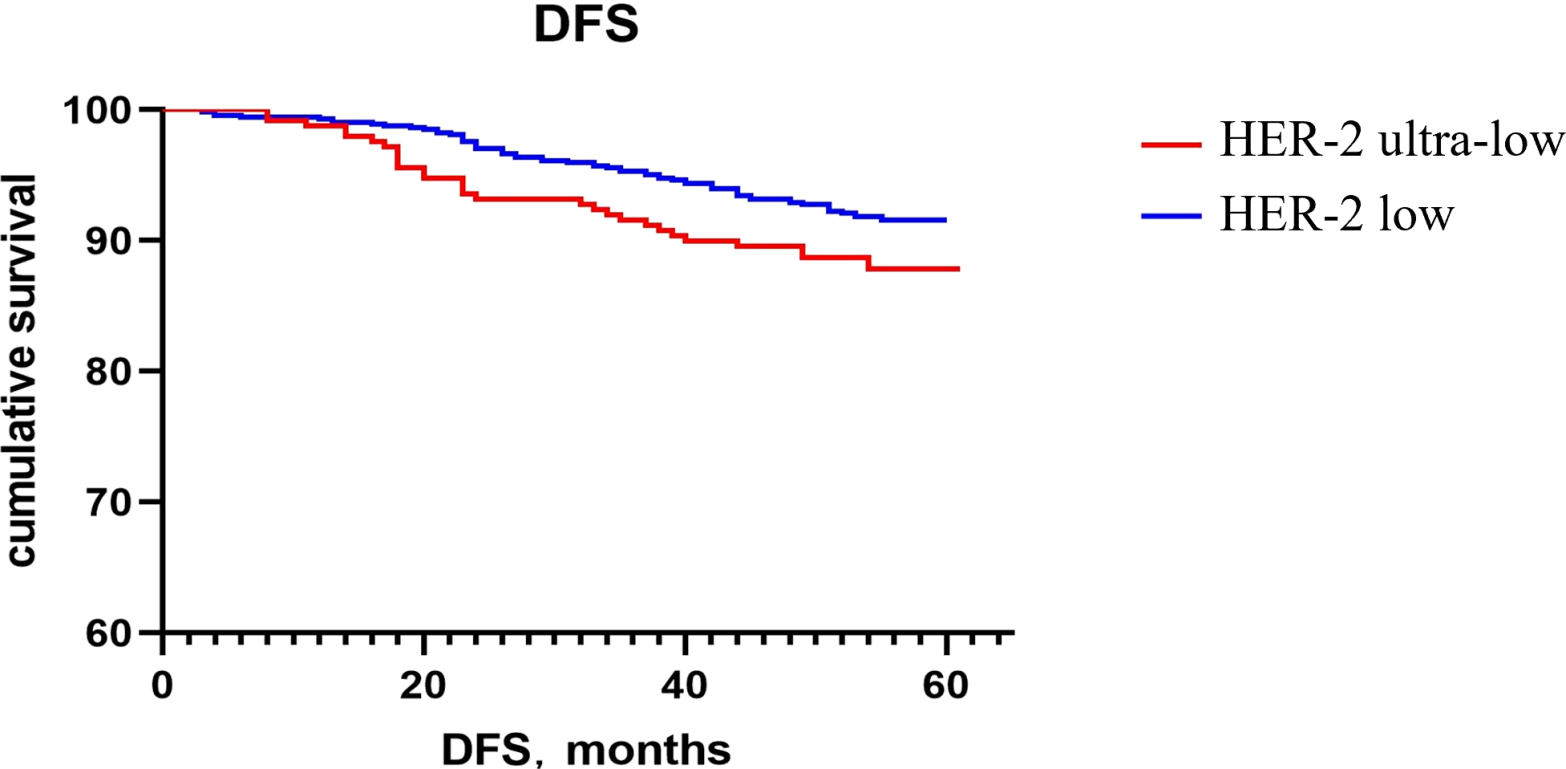
Comparison of the 5-year DFS rates between the HER-2 ultra-low and low expression groups.

**Figure 2:**
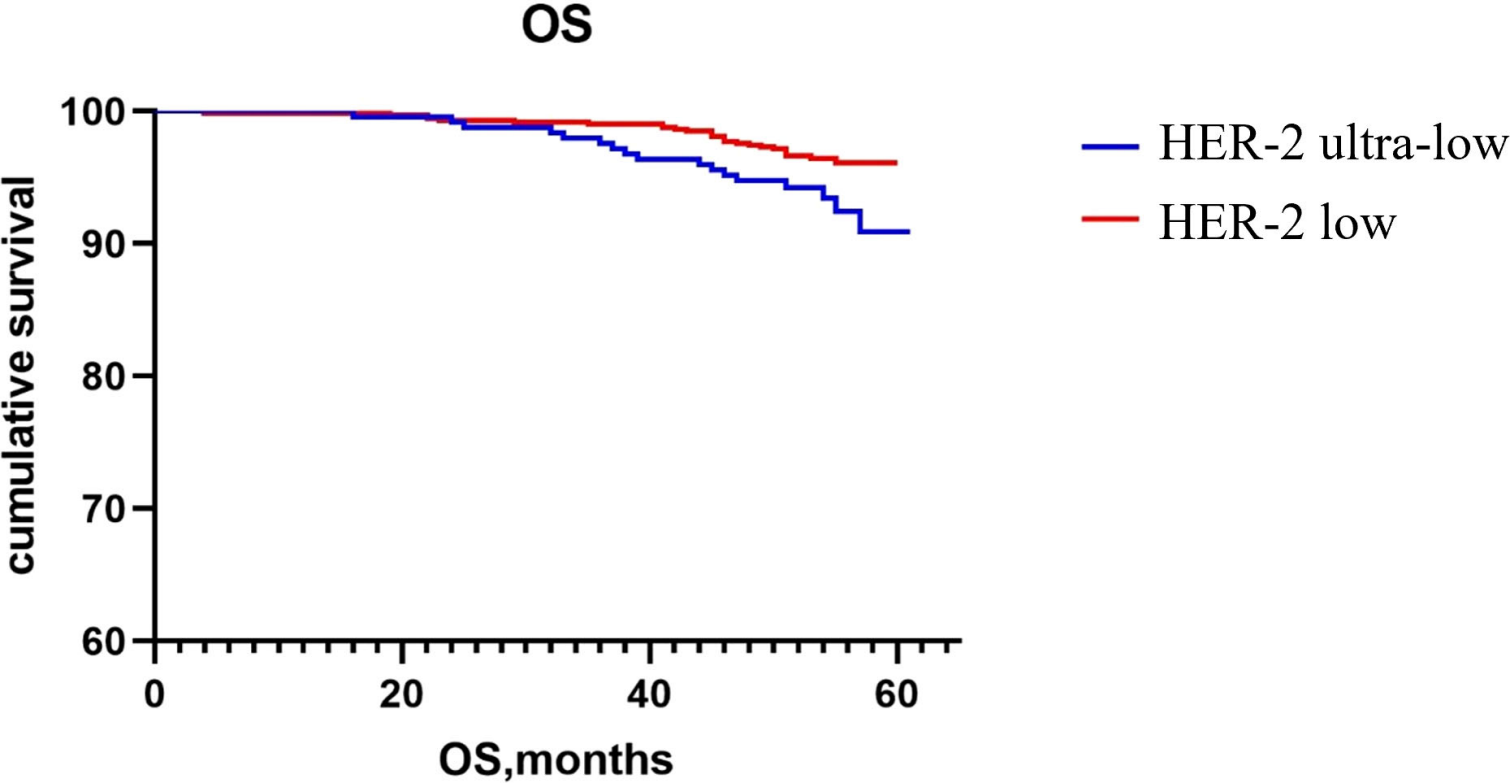
Comparison of 5-year OS rates between the HER-2 ultra-low and low expression groups.

The Kaplan–Meier method was used to generate the survival curve of the overall population. The 5-year DFS survival curve of the HER-2 ultra-low and low groups had a trend of separation, and the difference did not reach statistical significance (P = 0.068; **Figure 1**). The OS rate of HER-2 ultra-low group was lower than that of the HER-2 low group (P = 0.022; **Figure 2**).

The results of the univariate COX regression analysis showed that lymph node metastasis, tumor diameter, and Ki67 expression were risk factors affecting the 5-year DFS rates in patients with ultra-low HER-2 expression (P < 0.05; **Table 3**). Tumor diameter and lymph node metastasis were risk factors affecting the 5-year OS rate in patients with HER-2 ultra-low expression (P < 0.05; **Table 3**). Lymph node metastasis was considered an independent risk factor affecting the 5-year OS rate in patients with HER-2 ultra-low expression (P < 0.05; **Table 3**).

**Table 3:** COX regression analysis of factors influencing DFS and OS in HER-2 ultra-low expression.

Characteristics	DFS						OS					
	Univariate COX analyses			Multivariate COX analyses			Univariate COX analyses			Multivariate COX analyses		
	OR	95% CI	*P*	OR	95% CI	*P*	OR	95% CI	*P*	OR	95% CI	*P*
T	2.297	1.297~4.069	0.004	1.469	0.783~2.754	0.231	2.483	1.308~4.713	0.005	1.938	0.929~4.044	0.078
N	1.947	1.337~2.833	0.001	1.79	1.163~2.754	0.008	2.148	1.342~3.439	0.001	1.883	1.095~3.238	0.022
Ki67	3.231	1.124~9.287	0.029	3.15	1.084~9.153	0.035	2.138	0.612~7.471	0.234			
ER	0.598	0.282~1.265	0.179				0.703	0.260~1.902	0.488			
PR	0.676	0.325~1.405	0.294				0.81	0.308~2.129	0.669			
p53	0.832	0.368~1.879	0.658				0.424	0.121~1.479	0.178			
TOPOII	1.329	0.628~2.815	0.457				1.407	0.519~3.811	0.502			
Age	0.86	0.460~1.607	0.637				0.769	0.345~1.717	0.522			
Histological stage	0.828	0.528~1.298	0.41				0.774	0.429~1.395	0.394			

OR, odds ratio; CI, confidence interval.

## Discussion

The treatment landscape for breast cancer was positively impacted by the molecular classification of subtypes initially proposed by Professor Charles Perou in 2000: luminal (HR+), basal (HR− and HER-2−), and HER-2 overexpression (HR− and HER-2+). The work by Axel Ullrich et al. on HER-2 in 2019 opened new prospects in clinical research and oncology practice. HER-2 is a tyrosine kinase receptor of the human epidermal growth factor receptor family. Homo- or heterodimerization between receptors activates downstream signaling cascades, triggering the proliferation, migration, invasion, and survival of cancer cells. The extracellular domain (ECD) of HER-2 protein is composed of four subdomains: I, II, III, and IV. The therapeutic effects of several HER-2 directed monoclonal antibody drugs, such as trastuzumab, pertuzumab, and small molecule tyrosine kinase inhibitors (TKIs), mainly stem from interactions with the different domains of the ECD.

The Food and Drug Administration (FDA) approved trastuzumab in 1990 for the treatment of HER-2+ metastatic breast cancer. As the first humanized monoclonal antibody against HER-2, trastuzumab binds to the ECD IV of HER-2 (9), thereby inhibiting the downstream intracellular HER-2 signaling pathway, arresting the cell cycle, and mediating antibody-dependent cytotoxicity (10). Although use of trastuzumab has improved the treatment outcomes in HER-2+, a large number of patients still develop drug resistance and breast cancer recurrence. Early studies have shown that targeting multiple domains of HER-2 receptor can synergistically exert anti-tumor effects. Pertuzumab was thus developed as a second humanized HER-2 monoclonal antibody. Unlike trastuzumab. which binds to subdomain IV of HER-2 ECD, pertuzumab binds to subdomain II of the HER-2 ECD and inhibits heterodimerization between (1) HER-2 and HER-1 and (2) HER-3 and HER-4, thereby blocking downstream tumor signaling (11). In 2017, the FDA approved the combination of pertuzumab and trastuzumab for patients with HER-2+ early-stage breast cancer at a high risk of recurrence. The combination of trastuzumab and pertuzumab blocks the HER-2 signaling pathway, which has helped establish its first-line status in anti-HER-2+ breast cancer treatment. TKIs can bind to the ECD of HER-2 and block the adenosine triphosphate-binding site of tyrosine kinase, thereby blocking downstream signal transmission and inhibiting the proliferation of cancer cells (12). TKIs are low molecular weight agents that can penetrate the blood-brain barrier, which is more advantageous in preventing and treating brain metastasis (12). Lapatinib, pyrotinib, and tucatinib are some examples of TKIs.

In 2013, the FDA approved T-DM1 for the treatment of HER-2+ metastatic breast cancer, making it the first ADC-class drug for solid tumor treatment. The payload can be delivered directly to the target cancer cells. The development of ADC drugs has provided multi-line treatment options for metastatic HER-2+ breast cancer. T-DM1 is a specific, potent, and stable combination of trastuzumab and microtubule inhibitor DM1 (a maytansine derivative) joined by a stable MCC (maleimidocaproyl (mc) and maleimidomethyl cyclohexane-1-carboxylate) linker (13). T-DM1 monotherapy has replaced traditional chemotherapy combined with targeted therapy. It has shown a good survival benefit in patients who are refractory to trastuzumab and pertuzumab combination therapy in the advanced stage and who do not achieve a complete pathological response after neoadjuvant chemotherapy. As the second approved drug in the ADC class for breast cancer, T-DXd has a higher drug-to-antibody ratio than T-DM1, with a linker that can be cleaved. The payload of T-DXd is a TOPOI inhibitor having high membrane permeability. Irrespective of HER-2 expression, T-DXd can penetrate the cell membrane and kill the surrounding cancer cells, known as the bystander effect (14).

The DS8201-A-J101 study showed that in patients with advanced breast cancer having low HER-2 expression, T-DXd treatment resulted in an ORR of 37.0% and an mPFS of 11.1 months after a median of 7.5 lines of previous treatment. The median disease control time was 10.4 months (15). These findings led to the concept of HER-2 low expression. The TROPICS-02 study showed that in the population with low HER-2 expression, sacituzumab govitecan could significantly prolong PFS in patients. A subgroup analysis showed that sacituzumab govitecan improved the survival benefit of patients with low HER-2 expression and HER-2 non-expression, which was consistent with the study population (16). In addition, the results of DESTINY-Breast04 phase III clinical trial showed that the mPFS and OS of patients receiving T-DXd were significantly higher than those of patients receiving physician-chosen chemotherapy (6). Exploring T-DXd in the treatment of HER-2 low-expression breast cancer has become a topic of increased focus. Considering the clinical benefit of T-DXd in breast cancer with HER-2 low expression, the scope of anti-HER-2 treatment has been extended from HER-2+ to HER-2 low expression or ultra-low expression. To date, there are no guidelines to clearly define the subtypes of breast cancer with low HER-2 expression. The current and ongoing studies have adopted the definition of low HER-2 expression in breast cancer as having an IHC score of 1+ or 2+ and no amplification of the HER-2 gene based on ISH. Therefore, the accurate classification of low HER-2 expression mainly depends on the sensitivity and reliability of the detection method. Currently, two semi-quantitative methods are commonly used to determine the expression levels of HER-2 in clinical practice. However, both assays have limitations, leading to non-consensus in the classification of breast cancers with low HER-2 expression. More specifically, the results of IHC and ISH are influenced by various factors before and during the analysis. Currently, in the clinical set-up, formalin-fixed, paraffin-embedded tissue sections are used in IHC and ISH assays. Studies have shown that formalin fixation may result in the visualization of reduced protein expression; therefore, IHC and other detection methods may underestimate HER-2 protein expression in tissues (17). A study showed (18) that in a cohort of 500 samples, 28.0% of the samples were determined to be IHC 1+ or 2+ using the 4B5 antibody compared to 11.6% seen after using HercepTest™. Only 21.6% of patients classified as IHC 1+ or 2+ using the 4B5 antibody showed consistent results when tested using HercepTest. In addition, factors, such as antibody clone, enzyme activity, reaction time, temperature, and substrate concentration, have a significant impact on HER-2 staining intensity, thereby affecting the HER-2 test results (19).

With increasing research on the subtypes, breast cancer with low HER-2 expression constitutes a molecularly diverse and clinically heterogeneous group, including the majority of HR+ tumors. Horisawa et al. (20) found that 90.2% of tumors with low HER-2 expression were HR+, and Miglietta et al. (21) found that low HER-2 expression was more common in HR+ tumors than in triple-negative tumors, both in primary and recurrent or metastatic tumors. Compared to HER-2 0 or 1+ breast cancer types, HER-2 2+ and ISH− had significantly increased tumor size, lymph node positivity, high histological grade, and high Ki67 index. In a study on non-metastatic TNBC, 83.8% of the tumors were HER-2−, and only 16.2% of the tumors showed low HER-2 expression (22). A pooled analysis of 2310 patients with HER-2− breast cancer from four prospective clinical trials using neoadjuvant therapies showed that compared with HER-2− type, HER-2-low had a relatively lower histological grade (grade III) and lower Ki-67 score. In the present study, 672 (89.8%) cases were HR+ and 76 (10.2%) cases were triple-negative in patients with low HER-2 expression; 182 (73.1%) cases were HR+ positive and 67 (26.9%) cases were triple-negative in patients with ultra-low HER-2 expression. The majority of HER-2 low and ultra-low expression tumors were HR+. The study also found that the HER-2 ultra-low group had a relatively higher histological grade III and higher Ki67 score compared to the HER-2 low group.

The HER-2 low group has shown a higher OS rate in the present analysis. The genetic differences between low HER-2 expression and HER-2− tumors may be responsible for the clinicopathological diversity between the two groups. Schettini et al. (23)[24] reported that lumen-related genes, such as BCL2, BAG1, Forkhead box A1 (FOXA1), and ESR1, were significantly up-regulated in the HER-2 low expression tumors compared to HER-2− tumors. In contrast, basal and proliferation-related genes, such as CCNE1, MKI67, and EXO1, were significantly down-regulated. This study also quantified HER-2 gene amplification in 774 breast cancer patients using the next-generation sequencing assay and reported that patients with HER-2 overexpression or low expression had a significantly higher copy number amplification of other genes, including CDK12, retinoic acid receptor, alpha (RARA), and Speckle Type BTB/POZ Protein (SPOP), compared to HER-2− patients. This could be indicative of the different mutational profiles of these populations.

Currently, the poor prognosis of patients with HER-2+ breast cancer despite effective anti-HER-2 targeted therapy is a recognized fact; however, the prognosis of patients with low HER-2 expression remains inconclusive. Horisawa et al. (20) investigated 4918 breast cancer cases and found no statistically significant difference in the prognosis of patients with low HER-2 expression and HER-2−, regardless of the HR status. Gampenrieder et al. (24) analyzed data from 1729 patients with metastatic breast cancer and concluded that low HER-2 expression had no significant effect on the OS in HR+ or triple-negative subgroups compared to HER-2− subgroup. A retrospective study showed that in patients with high genomic risk, the OS and DFS rates of early breast cancer with low HER-2 expression are significantly higher than those of the HER-2− group (25). Rossi et al. (26) studied 1150 breast cancer patients and showed that the DFS of the patients with the HER-2 IHC 2+ and ISH− type had a worse prognosis compared to those with the HER-2 IHC 0 or 1+ type. Ignatov et al. (27) also reported that HER-2 2+ and FISH− were poor prognostic markers in patients. Further, large clinical studies are needed to evaluate the HER-2 intensity in patient prognosis. In this study, the subgroup analysis showed that there was no statistically significant difference in recurrence and metastasis rates between patients with ultra-low and low HER-2 expression regardless of the HR status. Although there was no significant difference in the 5-year DFS rates between the two groups, there was a trend of separation in the 5-year DFS rates between the HER-2 ultra-low and low groups, as indicated on the Kaplan–Meier survival curve. It may be related to fewer patients enrolled in the HER-2 ultra-low expression group and a shorter follow-up time. The 5-year OS of the HER-2 low group was higher than that of the ultra-low group, which may be related to the higher pathological stage of the HER-2 low group and a higher expression intensity of p53, TOPOII, and Ki67. Breast cancer with an ultra-low expression of HER-2 was found to be more aggressive and had a worse prognosis.

Surgery is also an important factor associated with HER-2 expression. Our results showed that breast-conserving surgery with ALND (3.2%) and breast-conserving surgery with SLNB (31.3%) was usually performed in the HER-2 ultra-low group. However, mastectomy with SLNB (40.1%) and modified radical mastectomy (41.2%) was usually performed in the HER-2 low group. Thus, we believed that HER-2 expression is related to the stage and grade of breast cancer. Stage and grade are important factors affecting surgery type.

A clinical study showed that T-DXd still had a certain ORR in patients with advanced breast cancer having an extremely low expression of HER-2, which was the first time that the concept of HER-2 ultra-low expression was proposed. At present, HER-2 ultra-low expression is still classified as HER-2− breast cancer, and there is a lack of relevant literature on breast cancer with HER-2 ultra-low expression. Based on our present retrospective analysis, there are some differences in the clinicopathological characteristics between HER-2 ultra-low and low expression cases. The 5-year OS rate of the HER-2 low expression group was significantly higher than that of the HER-2 ultra-low expression group. Whether HER-2 ultra-low expression breast cancer can be classified as a new subtype to distinguish from HER-2− breast cancer still needs to be supported by clinical evidence. The biological characteristics of breast cancer with low- or ultra-low HER-2 expression have not been elucidated. In the future, HER-2 testing may shift to focus on HER-2 low expression or ultra-low expression.

## Conclusions

There are differences in the clinicopathological features of breast cancer with HER-2 ultra-low and low expression types. HER-2 low expression was associated with more aggressive tumors and had a worse prognosis compared to HER-2 ultra-low expression. This study provides a reference to consider in the treatment of breast cancer with HER-2-low and -ultra-low expression. However, there are some limitations in our study. First, our study is a retrospective analysis. Insufficient sample size and statistical power may have biased the results. Second, patients were followed up through outpatient review, hospitalization, and telephone. Data on adjuvant treatment was not retrieved, and data on concomitant diseases were missing. These two factors may impact PFS and OS. Third, due to missing information, some indicators and difference of gene expression could not evaluate further, for example, TOPOII. More studies are still needed to study how these indicators and genes affect the prognosis and the mechanism. Finally, we only explored the characteristics of the population in China and did not consider people from other regions. Further studies are still needed to verify the characteristics of HER-2 ultra-low breast cancer.

## Data availability statement

The original contributions presented in the study are included in the article/supplementary material. Further inquiries can be directed to the corresponding author.

## Author contributions

CG performed study concept and design, revised the manuscript and make final approval of the version. JS and LZ performed IHC staining, analyzed data, interpretated result and wrote the manuscript. All authors contributed to the article and approved the submitted version.
